# Predictive validity of the identification of seniors at risk (ISAR) screening tool in a Turkish emergency department

**DOI:** 10.1186/s12877-026-07289-x

**Published:** 2026-03-06

**Authors:** Şakir Hakan Aksu, Fikret Bildik, Merve Yazla, Aslıhan Küçük Albayrak, Hacer Dogan Varan, Mehmet Ali Aslaner, İsa Kılıçaslan, Ayfer Keleş

**Affiliations:** 1 Department of Emergency Medicine, İzmir City Hospital, Sevket Ince Mah., 2148/11 Sk., Bayraklı, İzmir, 35540 Türkiye; 2 Department of Emergency Medicine, Ankara Etlik City Hospital, Ankara, Türkiye; 3 Department of Emergency Medicine, Ankara Gülhane Training and Research Hospital, Ankara, Türkiye; 4 Department of Internal Medicine, Division of Geriatrics, Gazi University, Ankara, Türkiye; 5 Department of Emergency Medicine, Gazi University, Ankara, Türkiye

**Keywords:** ISAR, Emergency department, Elderly patients, Adverse outcomes, Geriatric assessment, Turkish validation

## Abstract

**Background:**

Emergency departments are increasingly managing older patients who frequently present with multimorbidity and functional decline, placing them at increased risk for short and long-term adverse outcomes. Early identification of patients at increased risk is essential for appropriate risk stratification and care planning. The Identification of Seniors at Risk (ISAR) is a brief screening tool developed to identify older emergency department patients at risk of adverse outcomes; however, its predictive performance has not been formally evaluated in the Turkish emergency department setting. This study aimed to evaluate the predictive validity and reliability of the Turkish version of ISAR among older ED patients.

**Methods:**

This prospective cohort study included patients aged ≥ 65 years presenting to a tertiary level emergency department in Türkiye. The ISAR tool was translated using a forward-backward translation process and administered at index ED presentation. Adverse outcomes were assessed at 30 and 180 days and included death, unplanned hospitalization, functional decline, or transfer to a nursing home. Predictive performance was evaluated using receiver operating characteristic (ROC) analysis, and the optimal ISAR cut-off was determined based on ROC analyses.

**Results:**

Among 356 included patients (median age:73 years), 54.5% were classified as high-risk (ISAR ≥ 2). High-risk status was significantly associated with advancing age, higher comorbidity burden, greater triage severity, longer ED stays and higher rates of hospital admission. At 30 days, ISAR demonstrated good predictive performance for the composite adverse outcome, with an area under the curve (AUC) of 0.790 (95% CI 0.741–0.838). Using a cut-off of ISAR ≥ 2, sensitivity was 89.3% and specificity was 61.7%. At 180 days, predictive performance remained robust, with an AUC of 0.818 (95% CI 0.772–0.864), sensitivity of 80.6%, and specificity of 71.1%. Inclusion of emergency department readmissions in the composite outcome resulted in lower sensitivity and higher specificity at both time points.

**Conclusions:**

The Turkish version of the ISAR screening tool demonstrated acceptable predictive validity for identifying older emergency department patients at risk of adverse outcomes, with consistently high sensitivity and moderate specificity. ISAR may be useful as a rapid risk stratification tool in the emergency department but should not be used as a standalone instrument for clinical decision-making.

**Supplementary Information:**

The online version contains supplementary material available at 10.1186/s12877-026-07289-x.

## Introduction

The proportion of the elderly population is increasing rapidly worldwide, and the rate of individuals aged 65 and over in the total population is expected to rise to 16% by 2050 [1]. Emergency departments (EDs) are frequently visited by older adults due to acute medical conditions, chronic diseases, functional impairment, and social support needs. With advancing age, patients become increasingly prone to frailty and functional decline, which are strongly associated with adverse outcomes [2, 3]. Factors such as polypharmacy, multimorbidity and cognitive decline in geriatric patients complicate management processes in the emergency department, making hospitalization and discharge decisions particularly challenging for physicians [4].

To address these challenges, several screening tools have been developed to estimate the short and long term adverse outcomes among elderly ED patients. One of the most widely studied instruments is the Identification of Seniors At Risk (ISAR) screening tool, introduced by McCusker et al. in 1999. It was a brief, six-item questionnaire that can be administered at the bedside within minutes. A score of ≥ 2 has been shown to identify older adults at high risk of mortality, prolonged hospitalization, functional decline, or need for institutional care [5]. Early identification and risk stratification of high risk individuals can help to optimize hospital admissions, prevent unnecessary interventions and establish post-discharge geriatric care plans.

ISAR has been validated in many countries such as Germany, Italy, Canada and Portugal and its reliability has been tested in different populations [6–9].

Emergency department utilization patterns, healthcare system organization, and approaches to older adult care vary across countries. In Türkiye, older adults have a high level of emergency department utilization compared with many western healthcare systems, and post-discharge care is often supported by family members rather than formal geriatric services [10, 11]. Cultural perceptions of aging, illness, and healthcare utilization may further influence help-seeking behavior and clinical outcomes, potentially affecting the prognostic performance of brief risk screening tools. Although several studies conducted in Türkiye have examined frailty, functional status, or short screening instruments in older emergency department populations, none represent a formal diagnostic validation of the ISAR tool with assessment of predictive performance [4, 12, 13]. Consequently, the validity of ISAR in the Turkish emergency department setting remains insufficiently characterized.

The aim of this study was to assess the predictive validity of the Turkish adaptation of the ISAR screening tool and to examine sensitivity in predicting adverse outcomes in patients. Beyond providing a point-by-point diagnostic validation, this study also offers insight into the performance of the ISAR tool within a healthcare setting characterized by distinct cultural and social dynamics.

## Methods

### Study design and determination of the population

This study was designed as a single-center, prospective, observational cohort study. Patients aged 65 years and older who presented to the emergency department were included in the study. Prior to the study, approval was obtained from Gazi University Ethics Committee on 29 December 2020 with approval number 2020 − 721. The study was conducted between 1 January 2021 and 30 January 2021. During the 30-day study period, it was planned to evaluate all patients aged 65 years and older who presented to the emergency department every day of the week and every hour of the day. Inclusion criteria were the ability to undergo ISAR assessment, either by direct patient response or by reliable information obtained from a relative or caregiver. Patients were excluded from the study if they, their relatives or carers could not answer the questions, if they were admitted to the emergency department in cardiac arrest, if they had repeated admissions (only the first admissions were included in the study), if they could not be interviewed at the 30th and 180th day, if they did not agree to the informed consent form, if the data collection form was filled in incorrectly or incompletely (including missing ISAR or KATZ-ADL assessments or follow-up contact information). Accordingly, patients with missing baseline variables or missing follow-up data were excluded from the analysis, and no imputation methods were applied.

### Data collection and patient follow-up

Before the study started, all research assistant doctors working in the emergency department were informed about the purpose of the study, participant consent process, the content of the data collection form and how the data would be collected. The data collection forms for the study were kept in the triage room and attached to the patient files by paramedics while the patients were directed to the examination rooms. Patient recruitment was conducted consecutively on a 24-hour, 7-day basis throughout the study period. The study forms were completed by the research assistant doctors who evaluated the patient. The necessary demographic data, type of presentation to the emergency department, presenting complaint, triage category, comorbidities of the participants according to Charlson comorbidity index (CCI), emergency department processes, duration of the patient’s stay in the ED and outcome were recorded. ISAR forms were completed for study purpose only, and did not influence triage or disposition decisions. In our ED, triage is performed using a color-coded system in which green indicates non-urgent conditions, yellow indicates urgent but stable conditions, and red indicates immediately life-threatening conditions. Patient allocation and management were based on clinical presentation and vital signs, in accordance with routine ED practice.

During the emergency department examination, the ISAR test and the KATZ Activities of Daily Living scale, which is often used to assess functional competence in older people, were applied and recorded [14].

After the index emergency department visit, patients were contacted by telephone at 30 and 180 days to obtain information on adverse outcomes. In accordance with the original ISAR study, the primary outcome was defined as the occurrence of any of the following events and analysed as a composite endpoint: death, unplanned hospitalization, transfer to a nursing home, or functional decline. Emergency department readmission, although clinically relevant in emergency medicine, was not included in the primary composite outcome but was incorporated into an extended composite outcome and evaluated in secondary analyses. Follow-up assessments were conducted by a single trained researcher using structured telephone interviews with patients or their relatives/caregivers. Information regarding hospital admissions, emergency department readmissions, or institutional care was primarily self-reported and was cross-checked with available hospital records when possible. Functional status was reassessed using the Katz Activities of Daily Living Scale, and functional decline was defined as a decrease of at least one point in the Katz ADL score compared with baseline.

### Translation of ISAR screening tool to Turkish

The ISAR tool consists of six dichotomous items in its original form (Table 1). The translation process followed a standard forward–backward translation approach. First, the original English version was independently translated into Turkish by two bilingual clinicians with experience in emergency medicine and geriatric medicine. These translations were reviewed jointly to resolve minor wording differences and to ensure conceptual equivalence with the original items. The reconciled Turkish version was then back-translated into English by two independent bilingual translators of the initial translation process. The back-translated versions were compared with the original ISAR tool, and no substantive discrepancies affecting item meaning were identified. As a result, the final Turkish version was considered conceptually consistent with the original instrument. Each item was scored as 1 point for a “Yes” response (with a reverse scoring for item 4); patients with a total score of ≥ 2 were classified as high risk (Supplementary Table 1).

**Table 1:** ISAR screening tool in the original language

	Questions	Yes	No
1	Before the illness or injury that brought you to the Emergency, did you need someone to help you on a regular basis?	1	0
2	Since the illness or injury that brought you to the Emergency, have you needed more help than usual to take care of yourself?	1	0
3	Have you been hospitalized for one or more nights during the past six months?	1	0
4	In general, do you see well?	0	1
5	In general, do you have serious problems with your memory?	1	0
6	Do you take more than three medications every day?	1	0
Total			

### Statistics

MedCalc software (ver. 23.4.5) was used to calculate the sample size. Calculations were based on ROC curve analysis testing AUC against the null value of 0.50. Assuming an expected AUC of 0.65 based on prior studies, α = 0.05, power = 0.95, and an anticipated event rate of 30% (negative to positive ratio 2.33), the minimum required sample size was approximately 227 participants (68 events and 159 non-events) [6]. The final cohort (*n* = 356) therefore exceeded this requirement and provided adequate precision for estimation of AUC and operating characteristics. Data were analysed using SPSS (version 23.0). Descriptive statistics are presented as median, interquartile range for continuous variables and percentages for categorical variables. Mann-Whitney U test and Pearson chi-square test were used for differences between groups. Cronbach’s alpha was calculated as a descriptive measure of internal consistency. Given the multidimensional and formative nature of the ISAR screening tool, internal consistency coefficients were interpreted cautiously and were not considered a primary indicator of reliability. ROC analysis was performed to calculate the performance (AUC, sensitivity, specificity) of the ISAR score in predicting adverse outcomes at day 30 and day 180. A p value of ≤ 0.05 was determined to be significant.

## Results

**Fig. 1:**
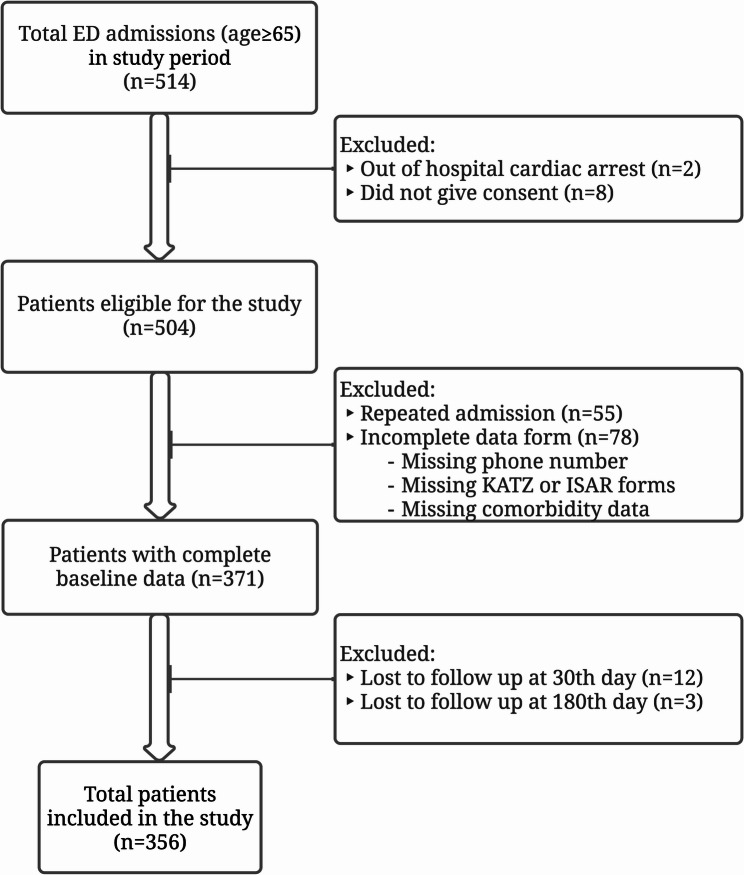
Flow diagram of patient inclusion and exclusion

During the 30-day study period, a total of 514 patients aged 65 years and older were admitted to the emergency department. After all exclusion criteria were applied, the study was conducted on the data of a total of 356 patients (Fig. 1).

### Patient characteristics

The median age of the participants was 73 years (range: 65–107), 52.2% were female, and the majority of patients (84.0%) presented to the emergency department by their own means of transport. The most common presenting complaint was gastrointestinal disorders (20.2%). According to emergency department triage coding 66.9% of patients were classified as green and 12.1% as red. In terms of disposition, 64% of patients were discharged, 18.5% were admitted to the ward, 6.7% were admitted to intensive care and 0.6% died in the ED (Table 3).

The Charlson comorbidity index scores were significantly higher in patients classified as high risk (ISAR ≥ 2) (Table 3). The most frequent comorbid conditions in the study population were diabetes (27.2%), previous myocardial infarction (21.1%), congestive heart failure (18.5%), chronic kidney disease (18.0%) and chronic lung disease (16%).

The prevalence of individual ISAR items varied from 21.3% to 50.0%. The most frequently endorsed item was polypharmacy (Q6, 50.0%), followed by increased need for help with self-care since the index illness (Q2, 45.2%), hospitalization in the past six months (Q3, 33.4%), and needing regular help prior to the index illness (Q1, 33.4%). The least frequently endorsed items were impaired vision (Q4, 21.9%), and serious problems with memory (Q5, 21.3%).

When evaluated with the ISAR screening tool, 54.5% (*n* = 194) of participants had a score ≥ 2, and 3.4% had the maximum score of 6. The distribution of total ISAR scores is presented in Table 2. The median ISAR score in the study population was 2 (IQR 0–4). When activities of daily living were assessed using the KATZ index, 63.8% of patients were unrestricted and 3.9% were dependent in all domains.

**Table 2:** Distribution of total ISAR scores in the study cohort

Total ISAR score	*N* (%)	Cumulative *N* (%)	*N* (%) ≥ this score
0	86 (24.2%)	86 (24.2%)	356 (100%)
1	76 (21.2%)	162 (45.5%)	270 (75.8%)
2	56 (15.7%)	218 (61.2%)	194 (54.5%)
3	57 (16.0%)	275 (77.2%)	138 (38.8%)
4	45 (12.6%)	320 (89.9%)	81 (22.8%)
5	24 (6.7%)	344 (96.6%)	36 (10.1%)
6	12 (3.4%)	356 (100%)	12 (3.4%)

Those who presented with general deterioration and respiratory symptoms were more likely to be in the high risk group (ISAR ≥ 2) than those who presented with other symptoms (*p* < 0.001). High-risk patients also had significantly longer ED stays and higher rates of ward and ICU admission (*p* < 0.001) (Table 3).

**Table 3:** Comparison of patient characteristics between groups according to ISAR score

		Total patients (*n* = 356)	ISAR < 2 (*n* = 162)	ISAR ≥ 2 (*n* = 194)	*p*
		Median (IQR) or *n*(%)*	Median (IQR) or *n*(%)	Median (IQR) or *n*(%)	
Age		73 (68–80)	71 (67–78)	75 (70–82)	<0.001
Age groups	65–74 ^*†*^	195 (54.8)	100 (51.3)	95 (48.7)	0.
	75–84	121 (34.0)	54 (44.6)	67 (55.4)	
	≥ 85 ^*†*^	40 (11.2)	8 (20.0)	32 (80)	
Gender	Female	186 (52.2)	85 (45.7)	101 (54.3)	0.939
	Male	170 (47.8)	77 (45.3)	93 (54.7)	
Marital status	Married	257 (72.2)	122 (47.5)	135 (52.5)	0.482
	Single	38 (10.7)	15 (39.5)	23 (60.5)	
	Widowed	61 (17.1)	25 (41.0)	36 (59.0)	
Living status	Home	342 (96.1)	160 (46.8)	182 (53.2)	0.017
	Other	14 (3.9)	2 (14.3)	12 (85.7)	
Arrival	Ambulance	57 (16.0)	18 (31.6)	39 (68.4)	0.021
	Own transport	299 (84.0)	144 (48.2)	155 (51.8)	
Application complaint	Gastrointestinal	72 (20.2)	36 (50)	36 (50.0)	
	Respiratory ^*†*^	61 (17.1)	7 (11.5)	54 (88.5)	
	Trauma ^*†*^	56 (15.7)	39 (69.6)	17 (30.4)	
	Neurological	52 (14.6)	31 (59.6)	21 (40.4)	
	Cardiovascular	32 (9)	17 (53.1)	15 (46.9)	
	Genitourinary	30 (8.4)	16 (53.3)	14 (46.7)	
	Musculoskeletal	29 (8.1)	13 (44.8)	16 (55.2)	
	Deterioration ^*†*^	24 (6.7)	3 (12.5)	21 (87.5)	
Triage code	Yellow	238 (66.9)	105 (44.1)	133 (55.9)	< 0.001
	Green ^*†*^	75 (21.1)	47 (62.7)	28 (37.3)	
	Red ^*†*^	43 (12.1)	10 (23.3)	33 (76.7)	
CCI	0–2	190 (53.4)	127 (66.8)	63 (33.2)	< 0.001
	≥ 3	166 (46.6)	35 (21.1)	131 (78.9)	
ED disposition	Discharged	228 (64)	118 (51.8)	110 (48.2)	< 0.001
	Admitted	66 (18.5)	24 (36.4)	42 (63.6)	
	LAMA	33 (9.3)	15 (45.5)	18 (54.5)	
	Admitted to ICU	24 (6.7)	4 (16.7)	20 (83.3)	
	IHT	3 (0.8)	1 (33.3)	2 (66.6)	
	Dead	2 (0.6)	0	2 (100)	
Length of stay	LOEDS (hrs)	4 (2–8)	3 (2–5)	5 (3-11.25)	< 0.001
	LOiHS (hrs)	170 (95–303)	123 (90.5-298.5)	205.5 (108–304)	0.209

*IQR* Interquartile range, *CCI* Charlson Comorbidity Index, *LAMA* Left against medical advice, *IHT* Interhospital transfer, *LOEDS* Length of emergency department stay, *LOiHS* Length of in-hospital stay

*: Shows column percentage

^**†**^Bonferroni adjusted *p* < 0.05

The proportion of patients classified as high-risk (ISAR ≥ 2) increased stepwise with advancing age, comorbidity burden, and triage severity. Post-hoc Bonferroni-adjusted analyses confirmed that these differences were primarily driven by the ≥ 85-year age group and higher triage severity. Among patients aged ≥ 85 years, 80% were high-risk compared with 49% of those aged 65–74 years (*p* = 0.001). Similarly, 78.9% of patients with a Charlson Comorbidity Index ≥ 3 were high-risk versus 33.2% with lower scores (*p* < 0.001). ISAR-positive patients were more likely to require admission to a ward or ICU and less likely to be discharged from the ED (*p* < 0.001). ICU admission was approximately four times more frequent among high-risk patients (10.3% vs. 2.5%). Median ED length of stay was also significantly longer in the high-risk group (5 h [IQR 3–11.25] vs. 3 h [IQR 2–5]; *p* < 0.001), whereas in-hospital length of stay showed a non-significant trend. Post-hoc Bonferroni-adjusted analyses showed that ISAR positivity was significantly more frequent among patients presenting with respiratory complaints, trauma, and general deterioration (Table 3).

**Fig. 2:**
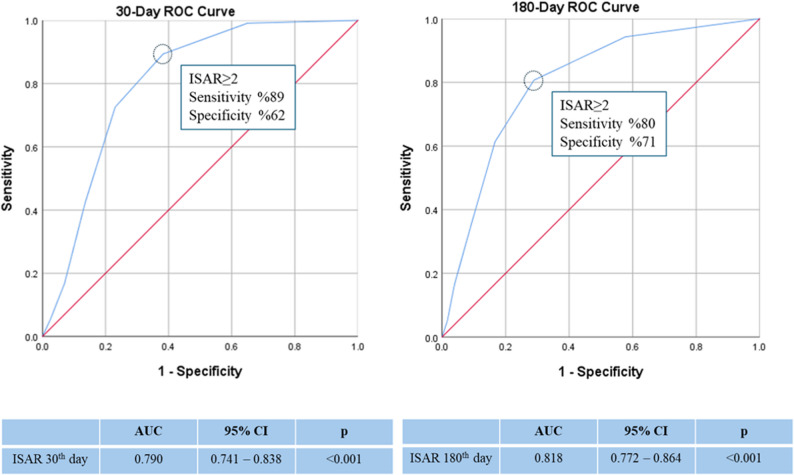
Receiver-operating characteristics (ROC) curve analysis for the ISAR score and combined end point at 30 days and 180 days

Table 4 summarizes the cumulative frequencies of individual adverse outcome components at 30 and 180 days. Overall composite adverse outcomes occurred in 31.5% and 49.4% at 30 and 180 days, respectively. ED readmission rates were frequent, whereas nursing home transfer remained uncommon at both time points. The predictive performance of ISAR is presented in Tables 5 and 6; Fig. 2. For the 30-day composite adverse outcome (mortality, unplanned hospitalization, or functional decline), the area under the curve (AUC) was 0.790 (95% CI: 0.741–0.838), indicating good predictive accuracy.

**Table 4:** Cumulative incidence of individual adverse outcome components at 30 and 180 days

Adverse Events	Day 30 (%)	Day 180 (%)
Functional decline	86 (24.1)	130 (36.5)
Transfer to a nursing home	2 (0.56)	13 (3.6)
Unplanned hospitalization	62 (17.4)	124 (34.8)
Death	23 (6.4)	56 (15.7)
Total adverse events #	113 (31.5)	176 (49.4)
ED readmission *	74 (20.7)	162 (45.5)

# Individual adverse outcome components may have occurred concurrently in the same patient

* ED readmission was not part of the primary composite adverse outcome and is reported separately for descriptive and secondary analyses

Using the cut-off of ISAR ≥ 2, sensitivity was 89.3% and specificity was 61.7%. At this threshold, ISAR demonstrated a high negative predictive value, supporting its utility as a screening tool for identifying patients at low risk of short-term adverse outcomes. Therefore, an ISAR score of ≥ 2 was used as the primary cut-off.

**Table 5:** Predictive performance of ISAR at different cut-off values for predicting the composite adverse outcome at 30 days

ISAR Cutoff points	Sensitivity (%95 CI)	Specificity (%95 CI)	PPV (%)	NPV (%)	LR+	LR-
≥ 1	99.12 95.17–99.98	34.98 (28.99–41.34)	41.5	98.8	1.52	0.03
≥ 2	89.38 82.18–94.39	61.73 (55.30–67.87)	52.1	92.6	2.34	0.17
≥ 3	72.57 (63.37–80.54)	76.95 (71.14–82.10)	59.4	85.8	3.15	0.36
≥ 4	42.48 (33.23–52.13)	86.42 (85.46–90.46)	59.3	76.4	3.13	0.67

*PPV* Positive predictive value, *NPV* Negative predictive value, *LR* Likelihood ratio

**Table 6:** Predictive performance of ISAR at different cut-off values for predicting the composite adverse outcome at 180 days

ISAR Cutoff points	Sensitivity (%95 CI)	Specificity (%95 CI)	PPV (%)	NPV (%)	LR+	LR-
≥ 1	94.32 89.90–97.24	42.22 (34.91–49.79)	61.5	88.4	1.63	0.13
≥ 2	80.68 74.06–86.24	71.11 (63.90–77.61)	73.2	79.0	2.79	0.27
≥ 3	61.71 (54.08–68.95)	83.33 (77.07–88.46)	78.3	68.8	3.70	0.46
≥ 4	36.36 (29.26–43.94)	90.56 (85.31–94.40)	79.0	59.3	3.85	0.70

*PPV* Positive predictive value, *NPV* Negative predictive value, *LR* Likelihood ratio

When emergency department readmissions were included in an extended composite outcome, sensitivity decreased to 82%, while specificity increased to 67%, reflecting the trade-off between sensitivity and specificity with broader outcome definitions.

At 180 days, ISAR showed slightly improved predictive performance, with an AUC of 0.818 (95% CI: 0.772–0.864). Using the same cut-off (ISAR ≥ 2), sensitivity was 80.6% and specificity was 71.1%. Inclusion of ED readmissions in the 180-day composite outcome further reduced sensitivity to 71% while increasing specificity to 73%. Likelihood ratios and predictive values across different ISAR cut-off points are detailed in Tables 5 and 6.

### Internal consistency

The internal consistency of the ISAR items, assessed using Cronbach’s alpha, was 0.69.

## Discussion

This prospective cohort study evaluated the validity and reliability of the Turkish version of the Identification of Seniors at Risk (ISAR) tool for predicting the 30- and 180-day composite adverse outcome in older emergency department (ED) patients. Using an ISAR cut-off of ≥ 2, the tool demonstrated high sensitivity and a high negative predictive value at both time points. These findings are consistent with ISAR functioning as a screening instrument that limits missed high-risk cases at the expense of over-triage, which aligns with its intended role in the ED setting.

Our findings are consistent with international research but also highlight important contextual differences. The original Canadian study reported sensitivity and specificity of 72% and 58% at the cut-off of ≥ 2 [5], whereas our cohort showed higher sensitivity at both follow-up horizons. This divergence may be more plausibly explained by structural and pathway-related factors. In some healthcare systems, a positive ISAR (or similar) screen triggers coordinated geriatric assessment, case management, or structured follow-up. Such downstream care may modify outcome rates and influence observed performance estimates. Across Turkey, ED-based risk screening does not routinely initiate a standardized geriatric pathway; similarly, in our setting, patients largely followed their natural clinical course after ED discharge [15]. Additionally, the limited availability of formal long-term care and community-based support in Turkey may increase reliance on hospital-based care among older adults, which could influence admission practices and downstream outcomes [16]. These contextual differences should therefore be considered when interpreting cross-study variation in ISAR performance.

Beyond predictive performance, the pattern of ISAR positivity across clinical subgroups supported the clinical coherence of the tool in this population. ISAR-positive classification clustered with expected markers of frailty and care complexity, including older age, higher comorbidity burden, higher triage acuity, and non-home living status, consistent with routine clinical expectations in the ED. These associations suggest that ISAR captures a clinically recognizable risk profile in our Turkish ED cohort.

Reporting score composition and item prevalence adds interpretive value beyond summary operating characteristics. First, the total-score distribution indicates that ISAR ≥ 2 identifies a sizeable segment of older ED patients (54.5%) as higher-risk, underscoring that implementation at this threshold should be framed as risk flagging rather than as a selective ‘rule-in’ strategy. Moreover, item prevalence did not suggest reliance on a single highly prevalent component that could artificially inflate positivity; instead, endorsement was distributed across domains, supporting the clinical interpretability of a multi-domain risk signal. In addition, the score distribution provides a practical perspective on threshold choice: moving from ≥ 2 to ≥ 3 would reclassify all patients with a score of exactly 2 (15.7% of the cohort) as screen-negative, shifting the balance toward specificity at the cost of potentially missed high-risk classifications. From a data-driven perspective, the ≥ 2 threshold was also supported by the Youden index in our cohort, while remaining consistent with ED screening priorities. On this basis, ISAR ≥ 2 is presented as a reasonable cut-off for Turkish ED practice.

Overall, our findings are broadly consistent with international ISAR validation studies from Germany [6], Italy [7] and Portugal [9], as well as prior work from Türkiye, underscoring how methodological differences shape reported operating characteristics. For example, functional decline was not included among adverse outcomes in the German and Portuguese studies, and outcome definitions and follow-up horizons varied across cohorts. In Türkiye, Sezik et al. reported moderate performance for 30-day outcomes using a higher threshold (≥ 3) and incorporating ED readmissions into the composite outcome [12], whereas Koçak et al. primarily evaluated frailty instruments in hospitalized patients with outcomes that are not directly comparable to ED-oriented composite adverse events [4]. Collectively, these comparisons highlight that differences in outcome composition, study population, and threshold selection can materially influence performance estimates across settings.

ISAR is best regarded as a pragmatic ED screening tool for short- and mid-term adverse outcomes rather than as a diagnostic frailty assessment. This positioning is supported by comparative evidence syntheses: a systematic review and meta-analysis emphasized ISAR’s role as a screening adjunct rather than a standalone decision instrument [17], and a systematic review reported that ISAR ranked among the more sensitive screening tools across evaluated instruments [18]. A prospective ED cohort study likewise reported supportive findings for ISAR predicting adverse outcomes in ED settings [19]. Accordingly, ISAR was not designed to replace formal frailty diagnostics or comprehensive geriatric assessment when these are specifically indicated; instead, it can function as an efficient “flag” to prioritize geriatric-oriented actions in the ED.

In light of our findings, we propose integrating ISAR into the Turkish ED workflow as a risk-flagging tool, capitalizing on its brevity for rapid application by triage nurses or first-contact physicians during the initial assessment. We suggest that an ISAR score of ≥ 2 triggers a defined geriatric-focused response. For patients being considered for discharge, a positive screen should prompt a structured review of social support and medication reconciliation to reduce preventable post-discharge adverse events. For patients being admitted, it can serve as an early signal to consider geriatric input and functional assessment – where available – such as inpatient geriatric consultation or comprehensive geriatric assessment (CGA), to address functional needs alongside acute medical stabilization.

## Limitations

This study has several limitations. First, it was conducted at a single tertiary care center that is frequently preferred by patients with complex and palliative conditions, which may limit the generalizability of the findings to other emergency department settings. Second, although an adequate sample size was achieved, the ISAR screening tool could only be completed in 69.2% of eligible geriatric admissions. Patients who did not complete the ISAR assessment may have represented a heterogeneous group, including both higher-acuity individuals and lower-acuity patients with brief emergency department encounters or early departure. In addition, a proportion of patients could not be reached at 30- or 180-day follow-up, which may have further contributed to selection bias. Consequently, the direction and magnitude of potential selection bias cannot be determined with certainty. Finally, while this study evaluated the predictive performance of the ISAR score for adverse outcomes, it did not assess its direct impact on clinical decision-making. Additionally, several ISAR items rely on patient and/or caregiver report; therefore, recall or reporting bias may have affected the accuracy of item responses and, consequently, ISAR classification in some participants. Future studies should explore how ISAR influences physician decisions and care pathways in routine emergency department practice.

## Conclusions

In conclusion, this study demonstrated that the Turkish version of the ISAR screening tool showed acceptable predictive validity for adverse outcomes among older adults presenting to the emergency department, with high sensitivity and moderate specificity at the ISAR ≥ 2 cut-off. The findings indicate that ISAR can help identify older emergency department patients at increased risk of adverse outcomes, although it should not be considered a standalone instrument for clinical decision-making. ISAR represents a brief and widely used screening tool that may support early risk stratification in the emergency department setting. Further multicenter studies are warranted to evaluate its diagnostic performance across different patient populations and to explore how ISAR may be incorporated into broader geriatric assessment pathways within healthcare systems.

## Supplementary Information

Supplementary Material 1.

## Data Availability

The datasets used and analysed during the current study are available from the corresponding author on reasonable request. Data are not available publicly because of privacy.
