# Supplementary material for: Predictive validity of the identification of seniors at risk (ISAR) screening tool in a Turkish emergency department
Source: BMC Geriatr. 2026 Mar 6;26:520. doi: 10.1186/s12877-026-07289-x (PMC13077872; doi:10.1186/s12877-026-07289-x)
Supplement: Supplementary file 1 — Supplementary Material 1. [file 12877_2026_7289_MOESM1_ESM.docx]

|  | Sorular | Evet | Hayır |
| --- | --- | --- | --- |
| 1 | Bu hastalık ya da yaralanma sizi acile getirmeden önce, gündelik hayatta herhangi birisinin yardımına ihtiyaç duyar mıydınız? | 1 | 0 |
| 2 | Bu hastalık ya da yaralanma sizi acile getirdiğinden beri, kendi bakımınız için normal hayatınıza göre daha fazla yardım ihtiyacı hisseder oldunuz mu? | 1 | 0 |
| 3 | Son 6 ayda 1 veya daha fazla gece hastanede yattınız mı? (acil servis başvuruları hariç) | 1 | 0 |
| 4 | Genelde görmeniz iyi midir? | 0 | 1 |
| 5 | Genelde hafızanızla ilgili ciddi sorunlar yaşar mısınız? | 1 | 0 |
| 6 | Günde 3’ten fazla farklı ilaç kullanıyor musunuz? | 1 | 0 |
| Toplam | |  |  |

Supplementary Table 1. Turkish translation of the ISAR screening tool
